# Noise Optimization Design of Frequency-Domain Air-Core Sensor Based on Capacitor Tuning Technology

**DOI:** 10.3390/s20010194

**Published:** 2019-12-29

**Authors:** Shengbao Yu, Yiming Wei, Jialin Zhang, Shilong Wang

**Affiliations:** College of Instrumentation & Electrical Engineering, Jilin University, Changchun 130061, China; yushengbao@jlu.edu.cn (S.Y.); weiym17@mails.jlu.edu.cn (Y.W.); zhangjl_100@163.com (J.Z.)

**Keywords:** frequency-domain electromagnetic method, coil sensor, low noise, amplification circuit

## Abstract

In the semi-aviation frequency-domain electromagnetic measurement, the induction air-core coil and the differential pre-amplifier circuit introduce noise, which affects the sensor and results in receiving weak signals and improving the signal-to-noise ratio of the system. In response to this problem, by analyzing the physical structure of the air-core coil sensor and the mechanism of the amplification circuit, combined with the simulation and experimental tests of voltage noise, current noise, resistance noise and other noise components, analyzed that the thermal noise is the main component of the sensor noise in the system frequency band, but directly removing the matching resistor increases the instability of the circuit, causes the coil to work in an underdamped state, and generates a time domain oscillation at the resonant frequency, source impedance analysis and analysis of differential pre-amplifier circuit in the frequency-domain detection method, abandoning the matching resistance scheme and magnetic flux negative feedback scheme. The matching capacitor is added to make the receiver detect the frequency range in the 1–10 kHz range. In normal operation, the noise level reaches 10 nV level, which not only increases the stability of the circuit, but also reduces the noise of the sensor. It has far-reaching significance for the detection of weak frequency signals.

## 1. Introduction

The frequency domain electromagnetic method is widely used in resource exploration and geological structure detection [1]. The semi-aviation frequency domain electromagnetic detection method can radiate alternating electromagnetic waves in earth and space by arranging an electric long wire source on the ground and outputting an alternating current to earth. During the electromagnetic wave propagation process, when there is an abnormal change in the electrical structure in the local area, the amplitude and phase of the propagation path and the electromagnetic field will also change accordingly. The receiving system collects signals in the air, and by identifying the abnormalities at different frequencies, information such as the position, depth, and size of the abnormal body can be determined. However, there is currently no commercial inductive magnetic sensor for the semi-aeronautical frequency domain electromagnetic detection system, so the development of magnetic sensors used in this field has an important role and significance.

Existing magnetic sensors for electromagnetic detection mainly include magnetic core rods and air-core coil sensors. Since the magnetic rod has a ferromagnetic core, the sensitivity is high, and it is easy to saturate when moving in the air, so the air-core is applied. The coil sensor performs semi-aero frequency domain electromagnetic detection. Air-core coil sensors are widely used in electromagnetic detection. The existing inductive air-core coil sensors commonly used for electromagnetic detection [2,3,4,5] mainly include: MTEM-AL from Canada Phoenix, and 3D-3 sensors from Canadian Geonics. Used for ground electromagnetic detection; Chen et al. analyzed the optimized design method of AEM sensor in detail, and developed a high-sensitivity airborne transient electromagnetic sensor with a diameter of 1.1 m and a resonant frequency of 35.6 kHz. Fu Lei et al. of Jilin University used magnetic flux negative feedback to reduce the resonant frequency noise of the sensor. The air-core coil sensor applied to the transient electromagnetic detection system was developed [6,7,8]. The overall noise is within 100 nV. The above sensors are used in various fields of electromagnetic detection. Because the sensors are used for the detection of electromagnetic signals, the noise and sensitivity of the sensors are the core technologies that determine the detection, and they are also the core problems of sensor applications at the domestic and international levels.

Based on the analysis of the noise source of the air-core coil sensor, in order to reduce the noise, by analyzing the physical structure of the air-core coil sensor and the mechanism of the amplifying circuit, combined with the experimental test, the thermal resistance of the matched resistor with a large proportion in the noise analysis is optimized. It is designed to achieve an overall noise of the coil sensor of up to the 10 nV level in the 1–10 kHz band. The experimental results show that the sensor has a good noise level, which is beneficial to semi-aeronautical frequency domain electromagnetic detection.

## 2. Air-Coil Sensor Model Analysis

**Figure 1:**
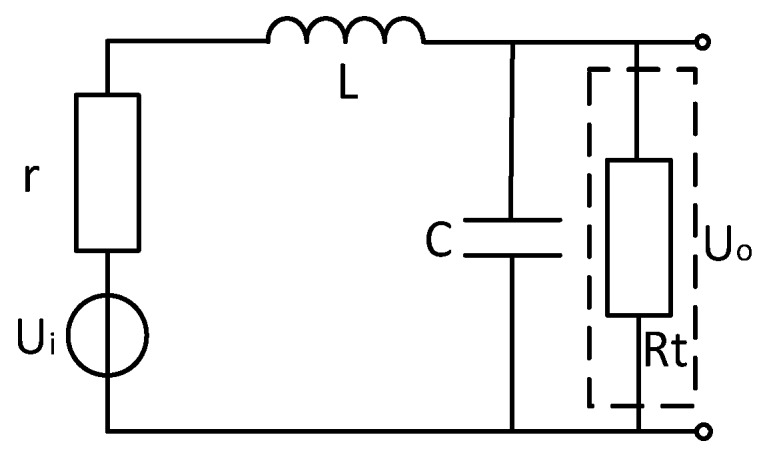
Air-coil sensor equivalent circuit.

The model of the traditional coil can be equivalent to the second-order system model of resistance, inductance and capacitance mixing [9,10,11,12], and its equivalent circuit is shown in Figure 1. In Figure 1: r is the internal resistance of the sensing coil; L is the equivalent inductance of the sensing coil; C is the equivalent capacitance of the sensing coil; Rt is the matching resistance of the sensing coil circuit; Ui is the theoretical induced voltage of the coil; Uo is the output voltage through the sensor coil; In order to achieve the near-damped state of the air-core coil, a matching resistor is usually used to match the sensing coil to reach the critical damping state., and the dotted line portion is a conventional coil matching resistor portion.

### 2.1. Air-Coil Parameter Design

The coil winding method, wire selection, skeleton diameter and other parameters will affect the resistance, inductance, and capacitance parameters of the sensing coil, and further affect the resonant frequency of the sensing coil. Therefore, it is meaningful to design a sensing coil that meets the requirements in combination with the detection requirements.

**Figure 2:**
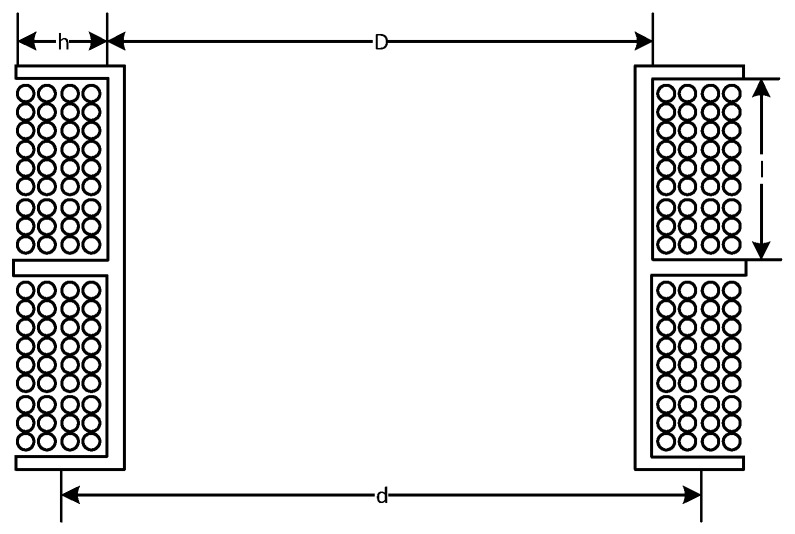
Air-core coil structure.

In Figure 2, D is the diameter of the coil, d is the average diameter of the coil winding, I is the width of each section of the coil, and h is the depth of each section of the coil.

(1) Air-coil sensor internal resistance

(1) $$r=\rho \frac{l}{S}=\frac{4\rho nd}{d_{w}^{2}}$$

where ρ is the resistivity of the enameled wire used, d is the average diameter of the coil, $d_{w}$ is the diameter of the wire, and n is the number of turns of the wire.

(2) Equivalent inductance of the air-coil

The inductance is related to the resonant frequency. The smaller the inductance, the larger the resonant frequency, so the inductance should be minimized.

(2) $$L=\frac{\mu_{0}dn^{2}}{4\pi }\left(\ln\left(\frac{8d}{b}\right)-1.73\right)$$

where b is the length of the coil wire cross section and μ_0_ is the permeability of the vacuum. Bring the relevant parameters of the sensor to obtain an equivalent inductance of 2.941 mH. The result measured by the Agilent 4263B model LRC tester was 2.907 mH, and the error between the simulation result and the measured result was 1.25%.

(3) Equivalent capacitance of the air-coil

The resonant frequency of the air-coil is inversely proportional to the distributed capacitance. In order to increase the resonant frequency, the distributed capacitance is reduced by segmented and layered coils. Therefore, the distributed capacitance of the actual coil is divided into interlayer capacitance and inter-segment capacitance.

In the air-coil winding process, the segmented structure (in two stages) can significantly reduce the distributed capacitance between the segments of the coil. The distributed capacitance between the segments of the coil is

(3) $$C_{g}=0.022\times \frac{4\left(N_{c}-2\right)}{N_{c}^{2}}\frac{\varepsilon_{g}l_{m}h}{e}$$

where $N_{c}$ is the number of layers of the coil, $\varepsilon_{g}$ is the relative dielectric constant of the skeleton, $l_{m}$ is the circumference of the coil, e is the inter-groove width of the bobbin, and h is the height of the bobbin.

When the coil winding method is the same, the distribution capacitance can be reduced by increasing the number of layers of the coil, but the inductance is inevitably increased, and the final result is that the resonant frequency of the coil increases. The interlayer distribution capacitance of the coil is

(4) $$C_{a}=0.118\times \frac{\varepsilon_{a}l_{m}l\left(N_{c}-1\right)}{N\delta N_{c}^{2}}$$

where $\varepsilon_{a}$ is the relative dielectric constant of the varnish of the enamel wire, l is the coil width, δ is the interlayer distance, and N is the number of coil segments. Bring the sensor parameters to obtain $C_{g}=4.89\mathrm{pF}$, $C_{a}=41.48\mathrm{pF}$, $C=C_{g}+C_{a}=46.37\mathrm{pF}$. It is calculated that the distributed capacitance between the sensor segments is smaller than the distribution capacitance between the layers, and the coil capacitance is 44 pF by the resonance method. The error between the two is 5.11%.

**Figure 3:**
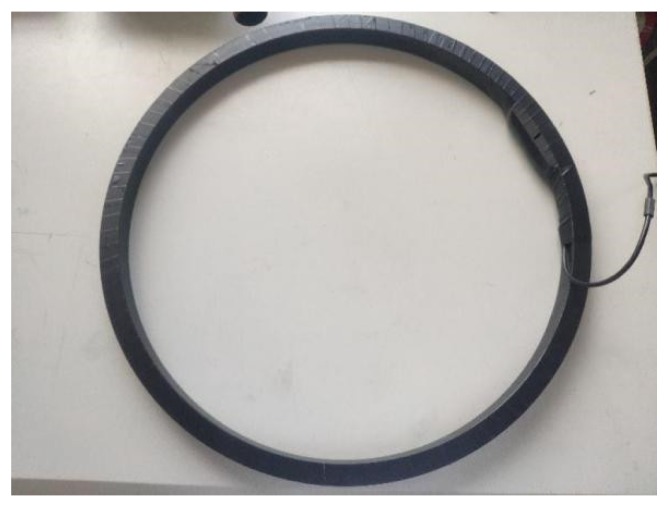
Air-coil.

The induction coil of this design is wound on a skeleton with a diameter of 0.5 m and is wound in two stages. Each section has ten layers (length), each layer is 6–7 inches (width), and the total number is 130 inches. After adding the amplifier, its equivalent area is 510 m^2^. The main parameters of the air-core coil are shown in Table 1. Figure 3 is a picture of the Air-coil.

### 2.2. Analysis of Frequency Characteristics of Air-Core Coil Sensor

The voltage generated by the inductive air-core coil sensor is derived from Faraday’s law of electromagnetic induction:

(5) $$V=-n\cdot \frac{d\phi }{dt}=-n\cdot S\cdot \frac{dB}{dt}$$

where: n is the number of sensing coil turns; φ is the magnetic flux passing through the coil; S is the average cross-sectional area of the coil; B is the magnetic induction.

It can be seen from the equivalent model of the coil that the voltage input to the amplifier is the voltage after the coil induced electromotive force passes through the equivalent model of the coil. According to the coil model of Figure 1, the transfer function of the coil is

(6) $$H\left(w\right)=\frac{U_{o}}{U_{i}}=\frac{1}{1+jwrC+jw\frac{L}{R_{t}}+\frac{r}{R_{t}}-w^{2}LC}$$

In the resistance matching mode, the damping coefficient of the coil is

(7) $$\xi =\frac{R_{t}rC+L}{2\sqrt{LCR_{t}\left(r+R_{t}\right)}}$$

The matching resistance of the coil is calculated as

(8) $$R_{t}=\frac{L}{2\xi \sqrt{LC}-rC}$$

**Figure 4:**
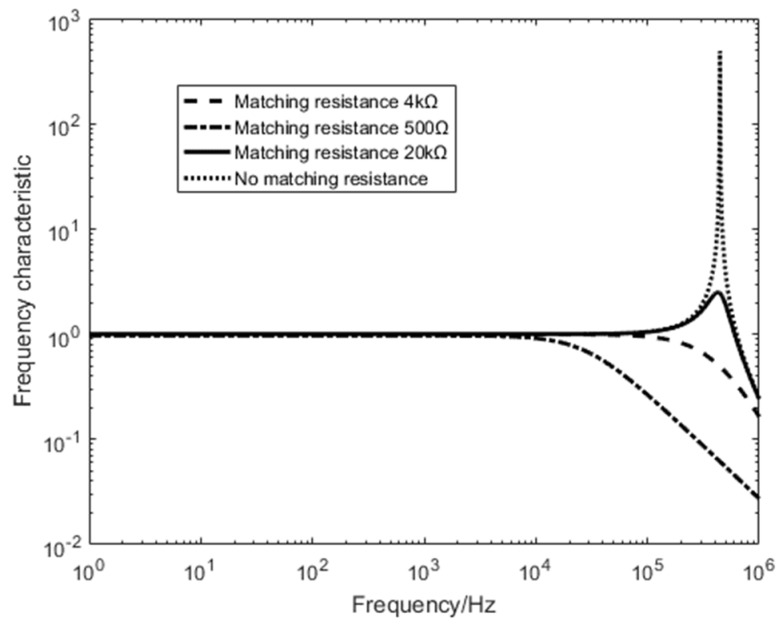
Sensor coil amplitude frequency characteristic diagram.

According to the formula, when $R_{t}=4k\Omega$, the damping coefficient is 1, the sensing coil is in the critical damping state, $R_{t}<4k\Omega$ is the overdamped state, and $R_{t}>4k\Omega \;$ is the underdamped state; the amplitude-frequency characteristics of the coil are shown in the Figure 4.

It can be seen from the Figure 4 that the sensitivity is higher when the coil is in critical damping and underdamping, because the frequency domain transient electromagnetic detection signal frequency range is 1–10 kHz, and the time domain oscillation caused by underdamping does not affect the frequency domain measurement. The system performs critical damping state (matching resistance is 4 kΩ) and no matching state (no matching resistance). The comparison test optimizes the working state and noise level of the sensing coil.

## 3. Analysis of Low Noise Operational Amplifier Model for Air-core Coil Sensor

**Figure 5:**
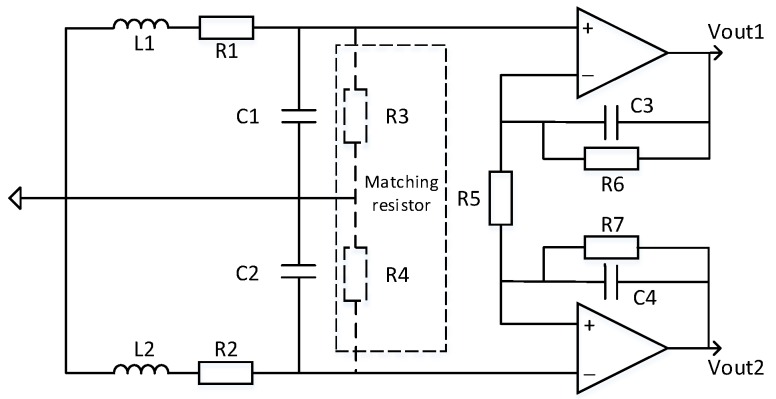
Sensor and op amp equivalent circuit diagram.

Since the noise of the preamplifier itself has strong interference with the transient electromagnetic signal, the self-noise should be minimized. The main noise source of the op amp is voltage noise and current noise. In the case of small impedance of the induction coil source at the front end of the circuit, this design uses the ultra-low noise amplifier LT1028 to amplify the received signal. The parameter of the amplifier is voltage noise $0.9\mathrm{nV}/{\mathrm{Hz}}^{1/2}$ and current noise $1\mathrm{pA}/{\mathrm{Hz}}^{1/2}$,this design uses differential amplification structure, and strictly symmetrical on the material selection of the device, reducing the DC offset caused by circuit asymmetry and reducing common mode noise. Therefore, the design circuit is shown in Figure 5.

## 4. Air-Coil Sensor Noise Analysis

The noise of the air-core coil sensor [13,14,15] is mainly divided into two categories: one is external noise, which is introduced from the external environment. External noise has sky noise, power line noise, etc., which cannot be completely eliminated. This paper uses differential amplification to reduce common mode noise. The other type is internal noise, mainly the thermal resistance noise in the coil and the noise of the preamplifier circuit. The internal noise has operational amplifier voltage noise, current noise, and resistance thermal noise, while the resistance thermal noise is further divided into matching resistance thermal noise and coil Thermal resistance noise, adjustable resistance thermal noise. In view of the existing problems of existing sensors, this paper analyzes the noise components, seeks noise reduction measures, and reduces the overall noise level of the sensors as much as possible.

### 4.1. Noise Analysis when Adding Matching Resistors (for Time-Domain)

**Figure 6:**
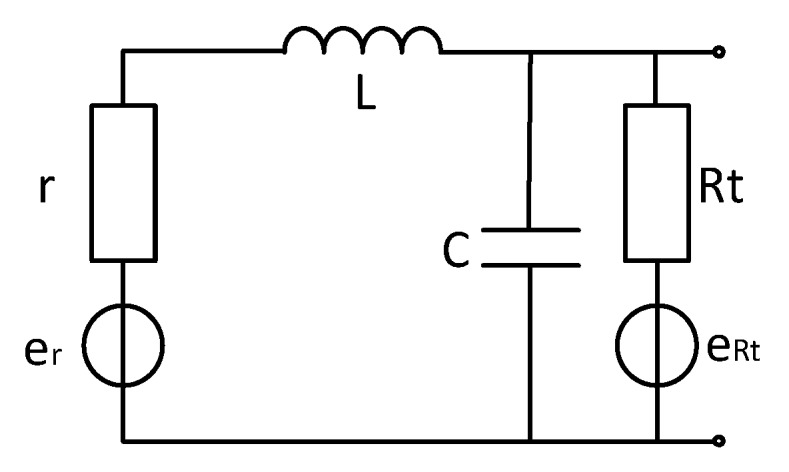
Air-coil model with matching resistor.

For the time domain electromagnetic method, the oscillation of the time domain signal should be avoided. The typical method is to make the circuit in critical damping state by means of resistance matching. Since the differential coil and single-ended coil analysis methods are roughly the same, this article starts with single-ended coil analysis. The coil model is shown in Figure 6.

According to the coil model, the transfer function of the coil is

(9) $$H\left(w\right)=\frac{1}{1+jwrC+jw\frac{L}{R_{t}}+\frac{r}{R_{t}}-w^{2}LC}$$

where: R_t_ is the matching resistance, r is the internal resistance of the coil, L is the inductance of the coil, and C is the coil capacitance.

(1) Op amp voltage noise

The equivalent noise e_no_ of the op amp voltage noise at the non-inverting input of the op amp is

(10) $$\left|e_{no}\right|={\left|e_{n}\right|}^{2}$$

where: en is the input voltage noise of the op amp.

Impulse current noise flowing through the coil generates voltage noise.

(2) Op amp current noise

The equivalent noise e_io_ of the op amp current noise flowing into the coil network at the non-inverting input of the op amp is

(11) $${\left|e_{io}\right|}^{2}={\left|i_{n}\right|}^{2}{\left|\frac{r+jwL}{1-w^{2}LC+jwrC+jw\frac{L}{R_{t}}+\frac{r}{R_{t}}}\right|}^{2}$$

where: i_n_ is the input current noise of the op amp.

(3) Thermal resistance in the coil

The equivalent noise e_r_ of the internal thermal resistance noise of the coil at the non-inverting input of the operational amplifier is

(12) $${\left|e_{r}\right|}^{2}={\left|\sqrt{4kTr}\right|}^{2}{\left|\frac{1}{1-w^{2}LC+jwrC+jw\frac{L}{R_{t}}+\frac{r}{R_{t}}}\right|}^{2}$$

where k is the Boltzmann constant, k = 1.38 × 10^−23^ J/K; T is the absolute temperature of the resistance in K.

(4) Op amp gain resistor and feedback resistor thermal noise

The thermal noise generated by the gain resistors R_1_ and R_2_ is equivalent to the equivalent noise e_Rq_ at the non-inverting input of the op amp.

(13) $${\left|e_{Rq}\right|}^{2}={\left|\sqrt{4kT\frac{R_{1}R_{2}}{\left(R_{1}+R_{2}\right)}}\right|}^{2}$$

(5) The voltage noise generated by the amplifier current noise flowing through the gain resistor

The equivalent noise e_iRq_ of the op amp current noise flowing through the gain resistor at the non-inverting input of the op amp is

(14) $${\left|e_{iRq}\right|}^{2}={\left|i_{n}\frac{R_{1}R_{2}}{\left(R_{1}+R_{2}\right)}\right|}^{2}$$

(6) Coil matching resistance thermal noise

The equivalent noise e_t_ of the coil matching resistor thermal noise at the non-inverting input of the op amp is

(15) $${\left|e_{t}\right|}^{2}={\left|\sqrt{\frac{4kT}{R_{t}}}\right|}^{2}{\left|\frac{r+jwL}{1-w^{2}LC+jwrC+jw\frac{L}{R_{t}}+\frac{r}{R_{t}}}\right|}^{2}$$

The total noise exit is

(16) $$\left|e_{nout}\right|={\left|e_{no}\right|}^{2}+{\left|e_{io}\right|}^{2}+{\left|e_{r}\right|}^{2}+{\left|e_{Rq}\right|}^{2}+{\left|e_{iRq}\right|}^{2}+{\left|e_{t}\right|}^{2}$$

The model uses an ultra-low noise amplifier to amplify the received signal. The gain resistance of the amplifier is R_1_ = 22.2 Ω, R_2_ = 200 Ω, R_t_ = 4000 Ω, filter capacitor 150 pF. The inductance of the induction coil is 2.907 mH, and the capacitance of the induction coil is 44 pF. The resonant frequency of the induction coil is 445 kHz.

**Figure 7:**
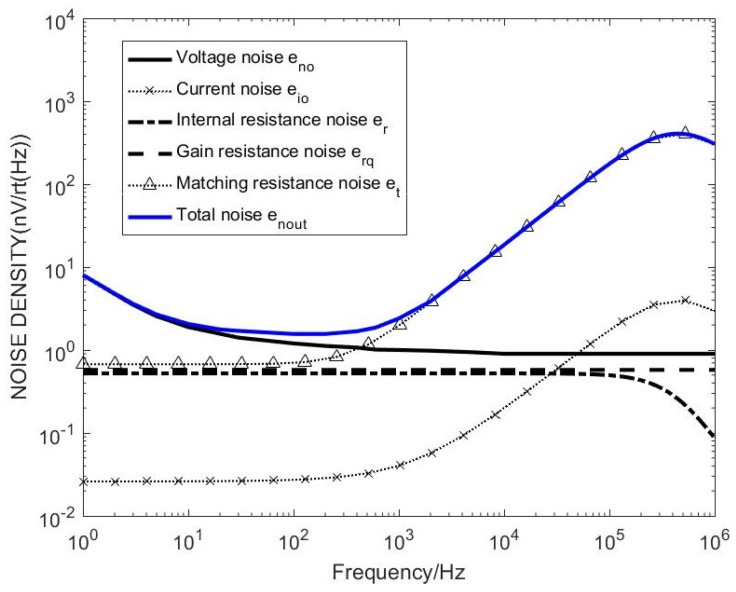
Noise distribution map with matching resistance sensor.

Combined with the coil model and the transfer function deduced in the paper, the distribution map of various noise sources can be obtained by bringing the unmatched circuit model into the model simulations, as shown in Figure 7.

It can be seen from Figure 7 that the matching resistor thermal noise affects the total noise of the sensor after adding the matching resistor, which has a larger effect than other noises. When the frequency is above 1 kHz, the current noise increases, and the matching resistor thermal noise increases, still contributing to the total. The main component of noise, the noise level reaches 100 nV level, affecting the global noise, so the most important consideration for reducing system noise is to reduce the matching resistance thermal noise.

### 4.2. Noise Analysis without Matching Resistors (for Frequency Domain)

**Figure 8:**
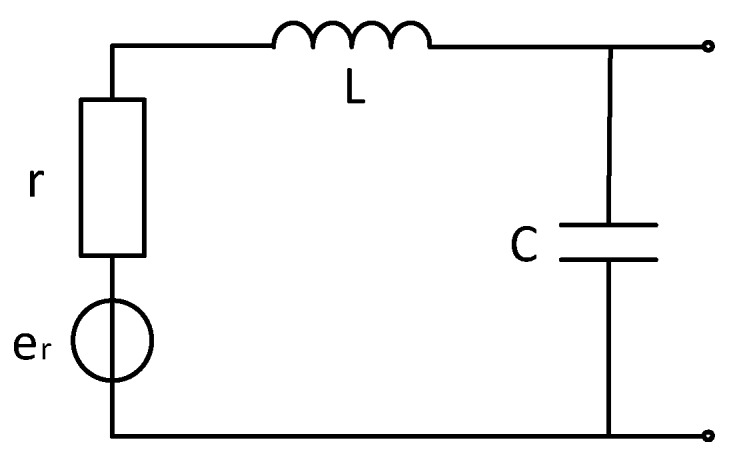
Coil model without matching resistor.

For the frequency domain electromagnetic method, the weak oscillation of the time domain signal does not affect the frequency domain signal measurement. When the coil resonance frequency is much higher than the frequency to be measured, it can be seen from the simulation analysis of the model in Section 4.1 that removing the traditional matching resistor can reduce the total system noise of the sensor in the effective frequency band. The coil model is shown in Figure 8.

According to the coil model, the transfer function of the coil is

(17) $$H\left(w\right)=\frac{1}{1+jwrC-w^{2}LC}$$

Combined with the unmatched circuit transfer function, all noise sources are represented as spectral density, and all noise is equivalent to the non-inverting input of the op amp for noise analysis.

(1) Op amp voltage noise

Equivalent noise of op amp voltage noise at the non-inverting input of the op amp is

(18) $$\left|e_{no}\right|={\left|e_{n}\right|}^{2}$$

where: e_n_ is the input voltage noise of the op amp.

(2) The voltage noise generated by the op amp current noise flowing through the coil network

The op amp current noise flows into the coil network to generate a noise voltage. The equivalent noise e_io_ at the non-inverting input of the op amp is

(19) $${\left|e_{io}\right|}^{2}={\left|i_{n}\right|}^{2}{\left|\frac{r+jwL}{1-w^{2}LC+jwrC}\right|}^{2}$$

where: i_n_ is the input current noise of the op amp.

(3) Thermal resistance noise inside the coil

The equivalent noise e_r_ of the internal thermal resistance noise of the coil at the non-inverting input of the operational amplifier is

(20) $${\left|e_{r}\right|}^{2}={\left|\sqrt{4kTr}\right|}^{2}{\left|\frac{1}{1-w^{2}LC+jwrC}\right|}^{2}$$

where k is the Boltzmann constant, k = 1.38 × 10^−23^ J/K;

(4) Op amp gain resistor and feedback resistor thermal noise

The thermal noise generated by the gain resistors R_1_ and R_2_ is equivalent to the equivalent noise e_Rq_ at the non-inverting input of the op amp.

(21) $${\left|e_{Rq}\right|}^{2}={\left|\sqrt{4kT\frac{R_{1}R_{2}}{\left(R_{1}+R_{2}\right)}}\right|}^{2}$$

(5) Voltage noise generated by the flow noise flowing through the gain resistor

The equivalent noise e_iRq_ of the op amp current noise flowing through the gain resistor at the non-inverting input of the op amp is

(22) $${\left|e_{iRq}\right|}^{2}={\left|i_{n}\frac{R_{1}R_{2}}{\left(R_{1}+R_{2}\right)}\right|}^{2}$$

The total noise e_nout_ is

(23) $$\left|e_{nout}\right|={\left|e_{no}\right|}^{2}+{\left|e_{io}\right|}^{2}+{\left|e_{r}\right|}^{2}+{\left|e_{Rq}\right|}^{2}+{\left|e_{iRq}\right|}^{2}$$

**Figure 9:**
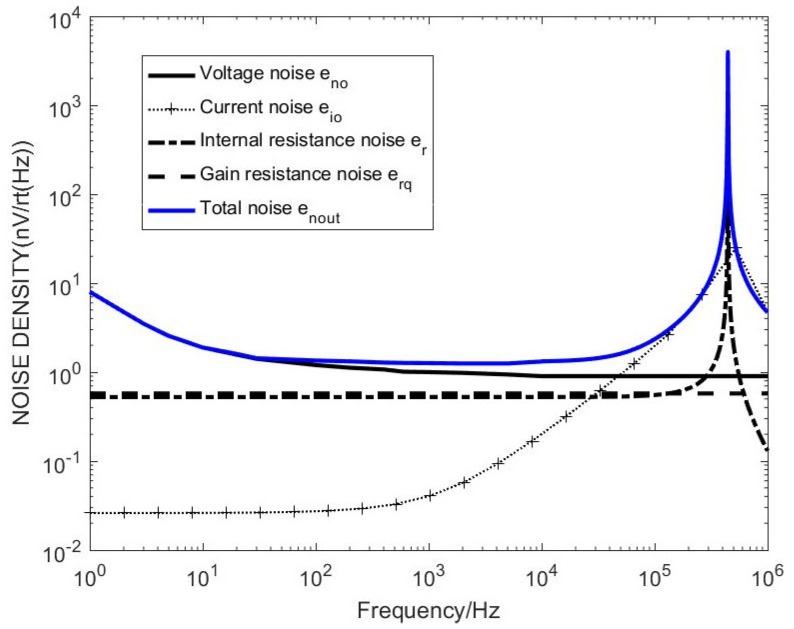
Noise distribution map without matching resistance sensor.

Combined with the coil model and the transfer function deduced in the paper, the distribution of various noise sources can be obtained by bringing the unmatched circuit model into the combined one with the coil model and the transfer function deduced in the paper, the distribution map of various noise sources can be obtained by bringing the unmatched circuit model into the model simulations, as shown in Figure 9.

It can be seen from Figure 9 that, after removing the matching resistor, the total noise of the sensor is significantly reduced around 1 kHz. The op amp voltage noise accounts for the largest proportion of the global noise before 10 kHz, which is limited by the low noise period and 1/f noise. The noise is reduced, the current noise increases with the increase of frequency, and the main component is occupied after 10 kHz. Since the system measurement frequency band is 1–10 kHz and the frequency is higher than 10 kHz, the noise is disturbed. Therefore, consider adding matching capacitors to adjust the resonance frequency.

### 4.3. Noise Analysis when Adding Matching Capacitors (for Frequency-Domain)

**Figure 10:**
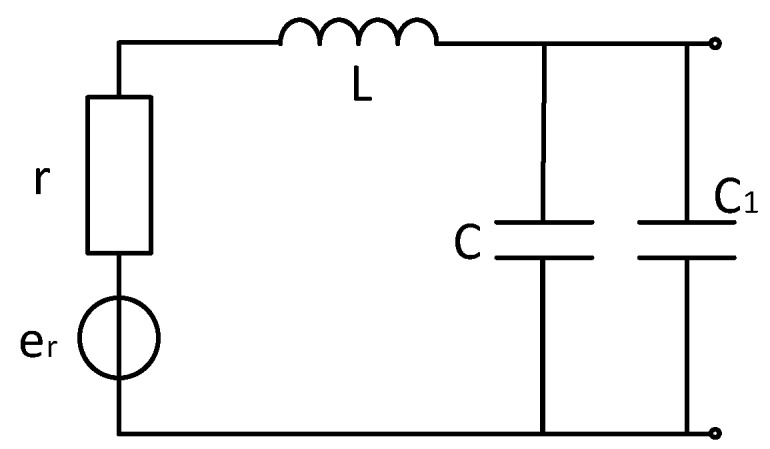
Noise distribution with matched capacitive sensor.

The coil model with matching capacitor is shown in Figure 10. According to the coil model, the coil transfer function is the same as the unmatched match. The difference is that the matching resistor is replaced with a matching capacitor. According to the total noise formula, the total noise is the sum of the squares of the noises of each part, because the voltage noise is inherent to the operational amplifier chip. The noise does not change because the capacitance of the matching capacitor changes, so use the formula

(24) $$\left|e_{nout}\right|={\left|e_{no}\right|}^{2}+{\left|e_{io}\right|}^{2}+{\left|e_{r}\right|}^{2}+{\left|e_{Rq}\right|}^{2}+{\left|e_{iRq}\right|}^{2}={\left|e_{no}\right|}^{2}+{\left|e_{1}\right|}^{2}$$

${\left|e_{1}\right|}^{2}$ is the square sum of noise other than voltage noise, it can be seen that as the capacitance of the matching capacitor increases, the resonant frequency of the sensor decreases, and the peak-to-peak value of the noise decreases at the resonant frequency. Since the receiving system has a detection frequency range of 1–10 kHz, it is necessary to ensure that no frequency oscillation (resonance noise) occurs in the re-band. As shown in the figure, the matching capacitor can meet the low noise requirement in the detection band when it is less than 15 nF. In order to reduce the resonance frequency noise value outside the detection band, the matching capacitor value should not be too small.

**Figure 11:**
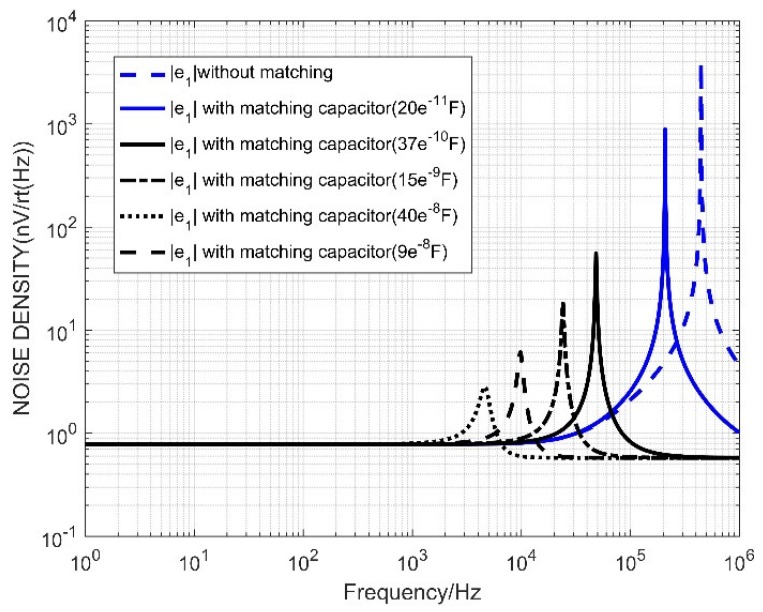
Noise distribution map with matched capacitive sensor.

The noise model simulation diagram is shown in Figure 11.

**Figure 12:**
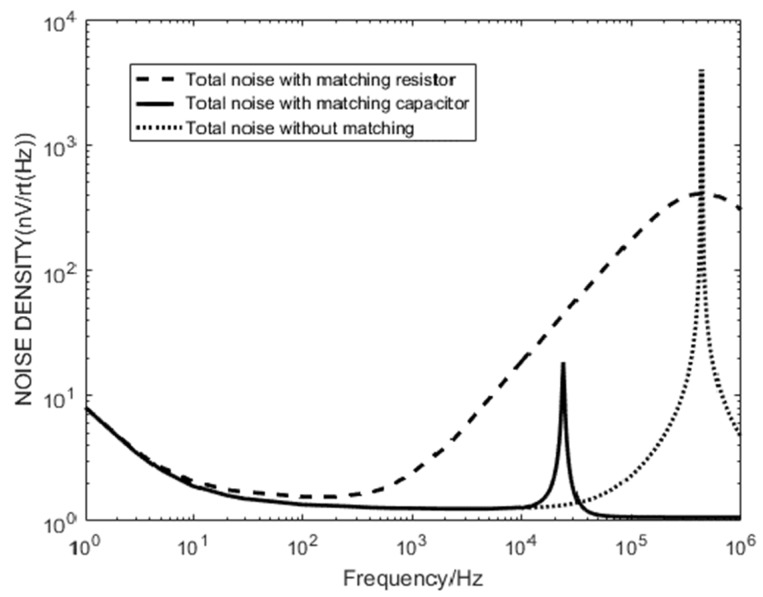
Comparison of sensor noise in three modes.

It can be seen that the matching capacitance 15 nF can meet the detection requirements. At this time, the noise is low and the noise band of the detection band is relatively flat. Comparing the first two matching methods, the system total noise simulation is performed, and the result is shown in Figure 12.

Resonant frequency adjustment by tuning the capacitor can suppress the total noise of the sensor and meet the requirements of detection stability, which has engineering application value.

## 5. Low Noise Sensor Noise Test and Analysis

**Figure 13:**
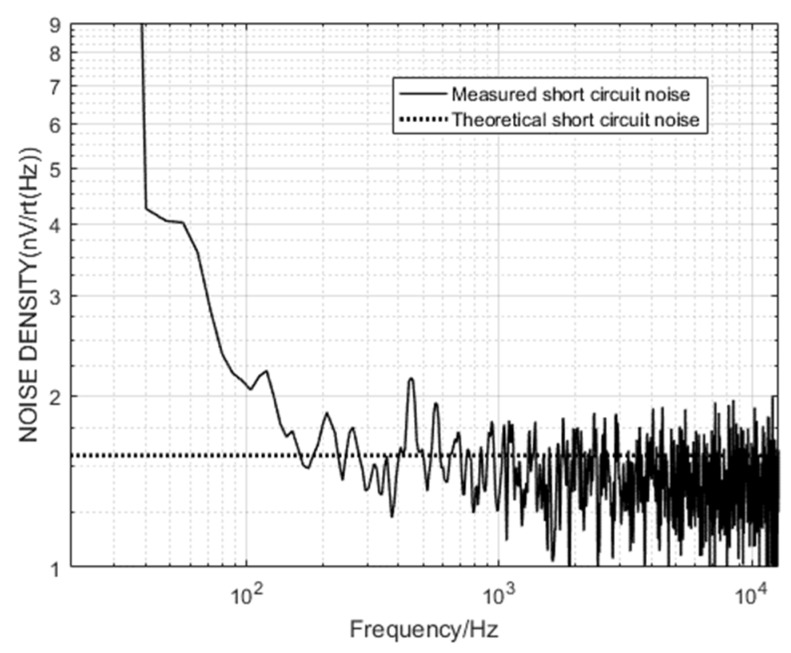
Sensor short-circuit noise measured map.

We place the sensor and amplifier circuit in the center of the electromagnetic shielding room for maximum shielding factor, the sensor input terminal is short-circuited, and the system short-circuit noise is measured by the dynamic signal analyzer 35670A. The comparison between the theoretical short-circuit noise and the actual short-circuit noise is shown in Figure 13.

According to the comparison, the input voltage noise of the 1/f region decreases with the increase of the frequency until reaching the corner frequency, and then the noise of the broadband voltage is low, and gradually increases when the frequency is greater than 10 kHz. Experimental and simulation results show that the sensor model has certain reliability.

**Figure 14:**
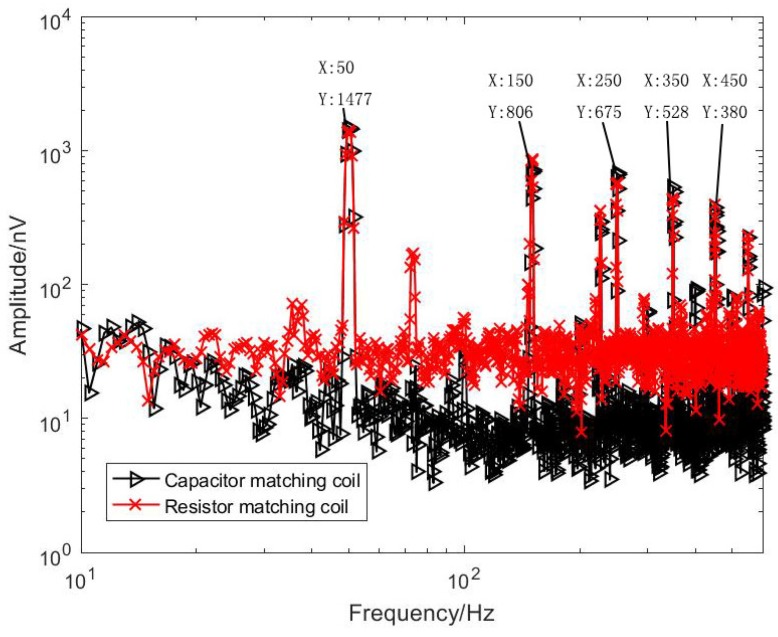
Two sensors measured data (test signal details).

**Figure 15:**
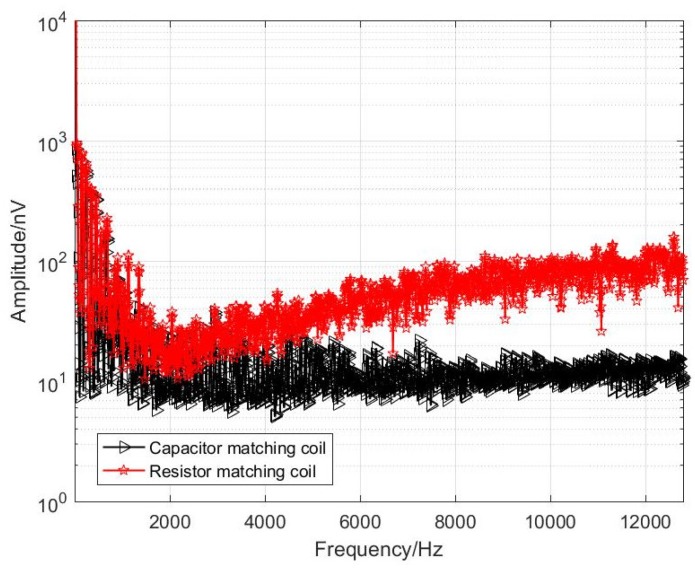
Two sensors measured data (entire frequency range of interest).

During the test, the sensor and the amplifying circuit are placed in the electromagnetic shielding room. Due to the large size of the sensor, the shielding effect cannot be put into the shielding cylinder. The shielding effect is not good, and it is limited by the magnetic shielding condition of the shielding room. There is power frequency noise interference. The equivalent input noise has some spikes at the power frequency. Therefore, the shielded indoor power frequency signal is tested as a low frequency to be the received signal (effective signal frequencies are 50 Hz, 150 Hz, 250 Hz, 350 Hz, 450 Hz). For comparison, the two sensor and amplifier circuit output signals were connected to the input of Agilent’s 35670A dynamic signal analyzer for simultaneous measurements. The comparison of the test results is shown in Figure 14 and Figure 15.

It can be seen from Figure 14 that, at the signal frequencies of 50 Hz, 150 Hz, 250 Hz, 350 Hz and 450 Hz, the optimized coil obtains a magnetic field signal with almost the same amplitude as that before the optimization, which is sufficient to explain that the homemade coil sensor measures the magnetic field signal accurately. Since the equivalent noise level of the unmatched coil is lower than that of the resistance-matched coil, the baseline of the signal measured by the unmatched sensor at the low frequency band is significantly lower than that of the resistance-matched sensor, reaching the level of 10 nV. As shown in Figure 15, the optimized coil noise performance is better in the 1–10 kHz band. In summary, for a semi-aeronautical frequency-domain electromagnetic detection system, the coil without matching resistors has a lower noise level for a low-frequency frequency-domain detection system and meets the detection requirements.

## 6. Conclusions

This paper analyzes the physical structure of the air-core coil sensor and the amplification circuit mechanism, and combines the noise components such as voltage noise, current noise, and resistance noise to perform modeling simulation and experimental testing. In order to solve the key noise sources, the thermal noise of the matching resistor, the method of removing the matching resistor and adding a matching capacitor are adopted to change the resonance frequency to reduce the noise at the resonance, increase the circuit stability and reduce the total system noise. The simulation and experiments verify that the optimized sensor in this paper has obtained consistent laboratory test results with the classic resistance matching sensor. The frequency point test results show that the capacitance matching sensor has obtained an effective signal of a nV-level effective magnetic field at the effective frequency and its original cost. The noise floor reaches a 10-nV level, and the noise is lower than that of resistance-matched sensors, which meets the actual detection requirements in the field. The optimized design scheme proposed in this paper meets the actual needs of the current group’s frequency domain electromagnetic detection system and enhances the system’s ability to detect weak signals.

## Figures and Tables

**Table 1:** Coil sensor parameters.

Parameter	Value
Number of coil segments (s)	2
Number of turns (n)	130
The average diameter (m)	0.46 m
Wire resistivity (ρ)	$1.7\times 10^{-8}\Omega /m$
Trunk width (e)	5 mm
Op amp voltage noise $\left(e_{n}\right)$	$0.9\mathrm{nV}/{\mathrm{Hz}}^{1/2}$
Op amp current noise $\left(i_{n}\right)$	$1.0\mathrm{pA}/{\mathrm{Hz}}^{1/2}$
